# An Extensible Positioning System for Locating Mobile Robots in Unfamiliar Environments

**DOI:** 10.3390/s19184025

**Published:** 2019-09-18

**Authors:** Xiaosu Xu, Xinghua Liu, Beichen Zhao, Bo Yang

**Affiliations:** 1 Key Laboratory of Micro-Inertial Instrument and Advanced Navigation Technology, Ministry of Education, Southeast University, Nanjing 210096, China; 2 School of Instrument Science and Engineering, Southeast University, Nanjing 210096, China

**Keywords:** extensible positioning system, mobile robot, unfamiliar environment, maximum correntropy Kalman filter, indoor positioning, data fusion

## Abstract

In this paper, an extensible positioning system for mobile robots is proposed. The system includes a stereo camera module, inertial measurement unit (IMU) and an ultra-wideband (UWB) network which includes five anchors, one of which is with the unknown position. The anchors in the positioning system are without requirements of communication between UWB anchors and without requirements of clock synchronization of the anchors. By locating the mobile robot using the original system, and then estimating the position of a new anchor using the ranging between the mobile robot and the new anchor, the system can be extended after adding the new anchor into the original system. In an unfamiliar environment (such as fire and other rescue sites), it is able to locate the mobile robot after extending itself. To add the new anchor into the positioning system, a recursive least squares (RLS) approach is used to estimate the position of the new anchor. A maximum correntropy Kalman filter (MCKF) which is based on the maximum correntropy criterion (MCC) is used to fuse data from the UWB network and IMU. The initial attitude of the mobile robot relative to the navigation frame is calculated though comparing position vectors given by a visual simultaneous localization and mapping (SLAM) system and the UWB system respectively. As shown in the experiment section, the root mean square error (RMSE) of the positioning result given by the proposed positioning system with all anchors is 0.130 m. In the unfamiliar environment, the RMSE is 0.131 m which is close to the RMSE (0.137 m) given by the original system with a difference of 0.006 m. Besides, the RMSE based on Euler distance of the new anchor is 0.061 m.

## 1. Introduction

The indoor positioning system (IPS) has been widely applied in many fields. Mobile robots, mine workers, indoor parking lots, factory cargo automation management, fire rescue, location detection of soldiers and so on rely on the support of indoor positioning technology. The existing indoor positioning technologies include Wi-Fi positioning, Bluetooth, ZigBee, RFID, Ultra-wideband (UWB) and so on [1]. UWB has become a good solution for high-precision indoor positioning technology due to its advantages of higher positioning accuracy, better penetration, and better confidentiality than other positioning methods [2]. UWB is a radio-based positioning technology. Its positioning principle can be divided into time of arrival (TOA), time difference of arrival (TDOA), angle of arrival (AOA) and received signal strength indication (RSSI) [3,4,5,6]. Because a single positioning scheme is unable to solve all the problems of indoor positioning completely, indoor integrated navigation positioning technologies have become one of the key research areas. Typical indoor positioning schemes include UWB and SINS (strap-down inertial navigation system) indoor integrated navigation [7,8,9], and UWB and visual simultaneous localization and mapping (SLAM) integrated navigation [10], and so on. In order to improve the robustness of the positioning system, some scholars proposed UWB, INS (inertial navigation system) and visual SLAM integrated navigation [11].

In the age of artificial intelligence, mobile robots are ubiquitous. Various kinds of mobile robots have been invented [12], and even quantum drones have been invented to try to construct quantum communication networks [13]. An important research topic of mobile robots is the location of mobile robots, especially indoor mobile robots. Outdoors, the global satellite positioning system (GNSS) can be used to locate mobile robots [14,15]. Alas, indoors it is hard to receive the signal of GNSS, so GNSS to locate the mobile robot is not able to be used. In order to locate indoor mobile robots, scholars have proposed many methods, including UWB positioning, inertial measurement unit (IMU) based inertial navigation positioning, vision-based SLAM positioning and multi-sensor fusion based combination positioning [16,17,18,19,20,21]. In [19], a Sage-Husa fuzzy adaptive filter is used to solve the time-varying noise problem. For mobile robots, accurate positioning is one of the most basic and important requirements. How to make mobile robots know their position accurately in an unfamiliar environment is also an important topic for researchers. In order to achieve this goal, a robust positioning system needs to be studied.

In this paper, we present an extensible positioning system to locate mobile robots in unfamiliar environments of mobile robots. The positioning system fuses multiple sensors, including UWB, IMU, camera and other sensors. The system can be expanded by adding new UWB anchors of which the positions are unknown, to locate mobile robots in an unfamiliar environment. By using this positioning system, when mobile robots move into an unfamiliar environment, it is able to locate mobile robots through the placement of new anchors.

Compared with the existing literature, the main contributions of this paper are summarized as follows:

(1) An extensible positioning system is proposed, which adds unknown UWB anchors to the existing positioning system. The UWB anchors do not need clock synchronization, and there is no need for communication between anchors.
(2) A positioning method for mobile robots is proposed. The proposed method based on multiple sensors fusion is available for a complex environment in which the information of the positioning system is incomplete.
(3) A real-time positioning system for collecting camera data, IMU data and UWB ranging data is built, and the maximum correlation entropy Kalman filter (MCKF) is used to fuse sensors’ data to locating mobile robots.
(4) Using the extended positioning system, the localization of the mobile robot in an unfamiliar environment is realized, and the feasibility and expansibility of the positioning system are verified.

After reviewing related work in Section 2, this article is structured as follows: In Section 3, we present the extensible positioning system. In particular, we introduce the platform of the positioning system in Section 3.1, introduce the positioning process and positioning method of the whole positioning system in Section 3.2, introduce the positioning method of INS subsystem and UWB subsystem in Section 3.3, establish the state-space model of the whole positioning system, use MCKF method for data fusion, and present the positioning method of mobile robot in Section 3.4, add unknown anchors to the existing system by the recursive least squares (RLS) method to expand the positioning system in Section 3.5. According to the method introduced in Section 3.4, we use the extended positioning system to locate the mobile robot in Section 3.6. In Section 4, we give the experimental verification for the method proposed in this paper. To give a reference path, while positioning the mobile robot using the extensible positioning system, we use a SLAM system to locate the mobile robot at the same time. The SLAM system is based on key-frame which is introduced in [21]. A stereo camera and an IMU which are integrated into one module is used to realize the SLAM system instead of a monocular camera. In Section 5, we summarize the work of this paper.

## 2. Related Work

The indoor positioning technologies of mobile robots include UWB technology, UWB/SINS integration technology, visual SLAM technology and so on. At present, indoor positioning technologies based on multi-sensors fusion are the trend of positioning technology. A simplified positioning model is shown in [22]. In [22], Dobrev realizes the 2-dimensional (2-D) position estimation and 1-D attitude estimation of the robot with UWB technology. Strohmeier estimates the attitude of the mobile robot based on UWB technology [16]. What is more, in Strohmeier’s method, he fuses data of a variety of sensors. Fan realizes the two-dimensional indoor positioning of mobile robots by tightly coupled UWB and IMU measurements [23]. Guo combines UWB and IMU technologies and implements cooperative relative localization (RL) of UAV using a 2-D strategy [24]. However, differing from Fan, Guo presents a method to locate multiple mobile robots at the same time, and when mobile robots move in 3-D space, the relative positions of multiple robots can be estimated in real-time. A 3-D positioning model is used to estimate the positions of mobile robots by Wang [25]. In this model, Wang uses a self-made structured light scanner to improve the positioning accuracy of the mobile robot. Mur-Artal and Tardos study the positioning technology of simultaneous localization and mapping (SLAM) and share their code online [20]. Based on the research of Mur-Artal and Tardos, scholars successfully apply ORB-SLAM2 to real-time positioning systems of mobile robots [26,27,28]. After that, SLAM technologies become a hot topic in the positioning of mobile robots. SLAM technologies deeply rely on the calculation speed of computers. In recent years, the advantages of visual-inertial systems (VINS) are found by Qin and other scholars. VINS is a multi-sensor system. Qin et al. applied VINS to the localization of mobile robots and realized the precise localization of mobile robots through visual relocation and Loop detection [21,29,30].

Data fusion of multi-sensor is an important part of multi-sensor integrated positioning technology. An effective technology of data fusion is Kalman filtering. Since the Kalman filter algorithm was proposed in 1960 [31], various improved algorithms based on Kalman filtering have been proposed. Based on the Kalman filter algorithm, some scholars proposed an extended Kalman filter (EKF). A typical description of the EKF is given in [32]. The EKF is a nonlinear filter, which is widely used in many fields. In recent years, nonlinear filters have been widely studied. Julier and Uhlmann proposed an unscented Kalman filter (UKF) in [33], Arulampalam proposed a particle filter (PF) in [34]. After that, a cubature Kalman filter (CKF) was proposed by Arasaratnam and Haykin in 2009 [35,36]. The CKF calculates the multi-dimensional weight integral with a spherical-radial rule and works well for high-order nonlinear systems. However, if the system model is inaccurate or the state of the system is abrupt, the CKF does not perform as well as expected. The KF, UKF, and CKF, etc. are all algorithms based on minimum mean square error (MMSE) criterion. If the model of the signal is non-Gaussian, the algorithms mentioned above are no longer applicable or ineffective. In order to solve the problem of the non-Gaussian signal model, Chen and Liu propose a filtering algorithm based on the maximum correntropy criterion (MCC) and called the maximum correntropy Kalman filter (MCKF) [37,38,39]. When the model of the signal is non-Gaussian, the filter works well, and when the model of the signal is a non-Gaussian model, the filter can work normally. The MCKF can also filter the coarse errors in the signal effectively.

Absolute positioning technologies using UWB measurements need to know the locations of the anchors before positioning. However, the measurement of a large number of anchors is a resource-intensive matter. Sometimes, the positioning environment is very specialized, for example, situations where the UWB ranging information is needed temporarily (such as unloading vehicles which are temporarily docked), and situations which are not enough time to measure the positions of all anchors in the UWB positioning system (such as fire and other rescue sites). In some environments, it is not possible to measure the locations of all anchors (such as a cavern). So, there is a need to study a technique to locate the anchors of the UWB positioning system. In [40], Mekonnen shows that the estimation of positions of anchors is a non-convex non-linear estimation problem. A few scholars have studied the self-localization problem of anchors [41,42]. They used the multidimensional scaling (MDS) approach and convex optimization methods to build an initial positioning system. The maximum likelihood (ML) estimator of the positions of anchors is relaxed to a semi-definite programming problem, so it is possible to calculate the positions of anchors. However, the existing self-localization methods of UWB anchors require communication between anchors, and clocks of anchors are required to be synchronous. When the anchors cannot communicate with each other, the positioning system is unable to establish, and the additional UWB anchors cannot be added to the positioning system. Therefore, it is still an unresolved and challenging problem to study the expansion of the positioning system when the clocks of independent anchors are not synchronized. The problem of locating mobile robots in an unfamiliar environment is still unsolved. In this paper, we propose an extensible positioning system, which aims at the absolute positioning of mobile robots in unfamiliar environments.

## 3. Extensible Positioning System

In this section, an extensible positioning system is proposed. Using an MCKF, the system fuses data from sensors such as UWB, IMU, camera and so on. In environments which mobile robots are unfamiliar with, the positioning system is expanded by adding new anchors through an RLS technic.

### 3.1. System Platform

**Figure 1:**
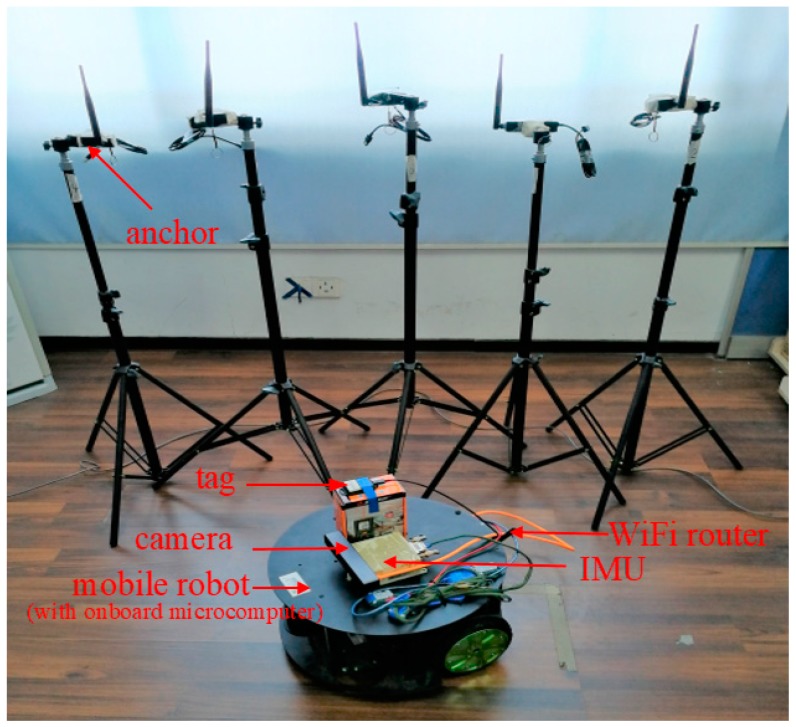
The platform of the positioning system.

As shown in Figure 1, the platform of the proposed positioning system includes a mobile robot and a location network which includes UWB, IMU, camera and so on. The mobile robot is an unmanned vehicle with a radius of 25 cm and a height of 25 cm. The mobile robot is equipped with an IMU, a UWB tag, a Stereo camera and a WiFi router. The onboard IMU includes a three-axis gyroscope and a three-axis accelerometer. The UWB tag is a part of a UWB system network. The tag on the mobile robot receives the ranging message of the UWB system. Another important part of the UWB system network is anchors. Anchors and the tag make up the entire UWB system which is a part of the location network. A microcomputer with a six-core CPU is used to control the mobile robot. Besides, a WiFi router is equipped on the mobile robot to transmit data between the mobile robot and the server in real-time. The detail of the platform is described as follows:

(1) IMU with a three-axis gyroscope and a three-axis accelerometer (BDStar Navigation: KY-INS110, Beijing, China)
(2) UWB transceiver with five anchors and a tag (DecaWave: DWM1000, Dublin, Ireland)
(3) Stereo camera (MYNTAI: MYNT EYE D1000-IR-120/Color, Jiangsu, China)
(4) WiFi router (gee routing: HC5661A)
(5) Onboard microcomputer (Rockchip: Firefly-RK3399, Fuzhou, China)

### 3.2. Positioning Process and Architecture of the System

**Figure 2:**
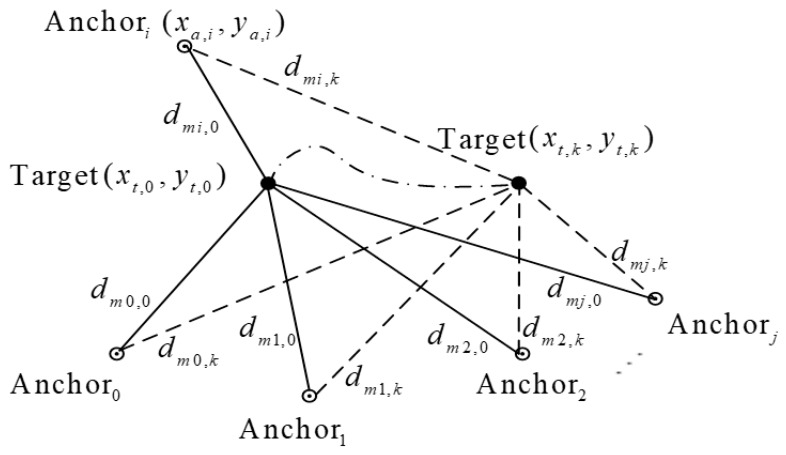
Positioning Process.

The positioning process of the system is shown in Figure 2. The entire positioning process includes four steps. In the first step, we use an existing positioning system which includes ${\mathrm{Anchor}}_{j}$ and tag to locate the mobile robot. In the second step, to extend our positioning system in unfamiliar environments, we calculate the position of unknown anchors (${\mathrm{Anchor}}_{i}$) and add those new anchors to our positioning system. For the third step, we use the extended positioning system to locate the mobile robot which carries the tag. In the last step, we describe how to use the positioning system to locate the mobile robot when it moves into an unfamiliar environment. The second step is the most important step in all of the steps. In the second step, the first thing to do is to put the tag on the mobile robot and then move the robot along a certain trajectory. The location $\left(x_{t,k},y_{t,k},z_{t,k}\right)$ of the tag is then determined based on the ranging measurements $d_{mj,k}$ between the tag and the anchors with known locations. During the movement of the mobile robot, the tag continuously communicates with anchors ${\mathrm{Anchor}}_{i}$ with unknown locations to obtain the ranging measurements $d_{mi,k}$. Then, based on the ranging measurements $d_{mi,k}$ and the estimated position $\left(x_{t,k},y_{t,k},z_{t,k}\right)$ of the tag on the mobile robot, the position $\left(x_{a,i},y_{a,i},z_{a,i}\right)$ of the unknown anchors can be estimated.

In this positioning system, we proposed an approach to estimate the position of the mobile robot using anchors with known positions. We also describe how new anchors with unknown positions are added to the existing positioning system to form an extension system. The process of locating a mobile robot using an extended positioning system is also described.

**Figure 3:**
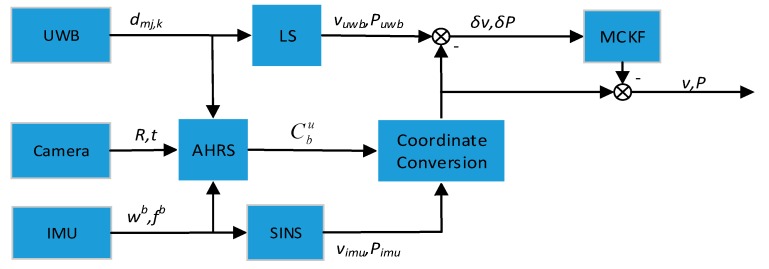
The Architecture of the positioning system.

The method of the positioning system is summarized in Figure 3. Based on the UWB measurements $d_{mj,k}$, IMU measurements $\left(\omega^{b},f^{b}\right)$ and camera measurements (R,t), the attitude matrix $C_{b}^{u}$ between the mobile robot frame (also called body frame) and the UWB system frame can be estimated using an attitude heading reference system (AHRS). Then, based on IMU measurements, the velocity $v_{imu}$ and the position $P_{imu}$ of the mobile robot relative to the body frame (b-frame) at the initial position can be estimated. At the same time, the velocity $v_{uwb}$ and position $P_{uwb}$ of the mobile robot in the UWB frame (u-frame, also the navigation frame in this paper) are calculated based on the measurements of the UWB system. Finally, an MCKF is used to fuse the two positions and velocities of the mobile robot to obtain the final position $v^{u}$ and velocity $P^{u}$ of the mobile robot.

### 3.3. SINS and UWB System

#### 3.3.1. Strapdown Inertial Navigation System

In the model of SINS, the acceleration and the angular velocity of the mobile robot in 3-D space are measured by the IMU. Equations of acceleration bias and gyroscope bias are as follows

(1) $${\dot{\nabla }}_{r}^{b}=-\frac{1}{\tau_{A}}\nabla_{r}^{b}+w_{rA}$$

(2) $${\dot{\varepsilon }}_{r}^{b}=-\frac{1}{\tau_{G}}\varepsilon_{r}^{b}+w_{rG}$$

where $\nabla_{r}^{b}$ is offset error vector of first-order Gauss–Markov process model of accelerators, $\varepsilon_{r}^{b}$ is offset error vector of gyroscopes, $w_{rA}$ and $w_{rG}$ are a white Gaussian noise (WGN) of $\nabla_{r}^{b}$ and $\varepsilon_{r}^{b}$ respectively. Thus, the IMU measurement model can be modeled by

(3) $${\tilde{f}}^{b}=f^{b}+\nabla_{r}^{b}+w_{A}$$

(4) $${\tilde{\omega }}^{b}=\omega^{b}+\varepsilon_{r}^{b}+w_{G}$$

where ${\tilde{f}}^{b}$ is the measurement vector of accelerators, ${\tilde{\omega }}^{b}$ is the output vector of gyroscopes, $f^{b}$ is the true value of specific force, $\omega^{b}$ is the true angle velocity of the mobile robot, $w_{A}$ and $w_{G}$ are a WGN of $f^{b}$ and $\omega^{b}$ respectively.

In a strap-down inertial navigation system, the kinematic equation of the mobile robot can be expressed as follows

(5) $${\dot{C}}_{b}^{u}=C_{b}^{u}(\omega_{ub}^{b}\times ),\omega_{ub}^{b}=\omega_{ib}^{b}-C_{u}^{b}(\omega_{ie}^{u}+\omega_{eu}^{u})$$

(6) $${\dot{v}}^{u}=C_{b}^{u}f^{b}-(2\omega_{ie}^{u}+\omega_{eu}^{u})\times v^{u}+g^{u}$$

where $C_{b}^{u}$ is the attitude matrix, $\omega_{ub}^{b}\times$ is a skew-symmetric matrix constructed with elements of angular velocity vector relative to the UWB frame, $\omega_{ib}^{b}$ is angular velocity of the mobile robot relative to the inertial frame, $\omega_{ie}^{u}$ is the Earth rotation velocity expressed in u-frame, $\omega_{eu}^{u}$ is the angular velocity of u-frame respect to Earth frame (e-frame) expressed in u-frame which is a zero vector in this model, $g^{u}$ is the gravity vector. $(2\omega_{ie}^{u}+\omega_{eu}^{u})\times v^{u}$ and $C_{u}^{b}(\omega_{ie}^{u}+\omega_{eu}^{u})$ are too small, they are considered as parts of the noise in our system. Therefore, Equations (5) and (6) can be simplified to

(7) $$\left\{ \begin{array}{l} {\dot{C}}_{b}^{u}=C_{b}^{u}(\omega_{ub}^{b}\times ),\omega_{ub}^{b}=\omega_{ib}^{b}=\omega^{b}\\ {\dot{v}}^{u}=C_{b}^{u}f^{b}+g^{u} \end{array}\right.$$

Then, using the simplified model, the velocity ${\hat{v}}_{imu}$ and position ${\hat{P}}_{imu}$ of the mobile robot can be obtained.

#### 3.3.2. UWB Positioning System Model

In dynamic positioning, the TOA method is more advantageous than the TDOA method [43]. So, in our system, we use the TOA method to estimate the position of the mobile robot. For simplicity, we use a least-squares (LS) method to estimate the position of the mobile robot. Following this, differentiate the position ${\hat{P}}_{uwb}$ to get the velocity ${\hat{v}}_{uwb}$. We assume that the UWB positioning system model can be described as

(8) $$\left\{ \begin{array}{l} {\hat{P}}_{uwb}=P_{uwb}-\delta P_{uwb}\\ {\hat{v}}_{uwb}=v_{uwb}-\delta v_{uwb} \end{array}\right.$$

where $\delta v_{uwb}$ and $\delta P_{uwb}$ are error vectors of the velocity and position respectively. Since the height of the mobile robot hardly changes, we consider its height to be a constant (the error of height is recorded as δz). The estimated position of the UWB system contains a large error, which causes a large error to be introduced when calculating the velocity. We assume that the motion model of the mobile robot is an even variable motion model and then use multiple positions estimates to calculate the velocity.

### 3.4. Data Fusion Based on MCKF

Data fusion of the positioning system is one of the most important parts of the positioning. Considering that the measurement errors of the IMU and UWB are everywhere when positioning, it is necessary to estimate the most possible position of the mobile robot. Measurement errors of IMU and UWB measurements cause positioning errors, especially coarse errors. To mitigate the effects of measurement errors on the positioning system, we used MCKF to fuse data from SINS and UWB systems. The model of MCKF is described as follows.

#### 3.4.1. State Space Model

An estimate of the position and velocity of the mobile robot is given by the UWB system and SINS respectively. According to the model of UWB positioning system and SINS, the state-space model of the positioning system can be described as

(9) $$\dot{X}=FX+GW$$

(10) $$Z=\left[\begin{array}{c} {\hat{v}}_{imu}-{\hat{v}}_{uwb}\\ {\hat{P}}_{imu}-{\hat{P}}_{uwb} \end{array}\right]=HX+V$$

where the state vector $X={\left[\begin{array}{ccccc} {\left(\delta \phi \right)}^{T} & {\left(\delta v\right)}^{T} & {\left(\delta P\right)}^{T} & {\left(\varepsilon_{r}^{b}\right)}^{T} & {\left(\nabla_{r}^{b}\right)}^{T} \end{array}\right]}^{T}$ is a 15-dimensional state vector, δϕ is an error vector of Euler angle, the velocity error δv is the difference between ${\hat{v}}_{imu}$ and ${\hat{v}}_{uwb}$, the position error δP represents the difference between ${\hat{P}}_{imu}$ and ${\hat{P}}_{uwb}$, V is the observation noise vector of the system, W is the systematic error which is a WGN. The state-space model is similar to the model of SINS and GPS integrated navigation [44], but not the same, especially the meaning of the measurements.

#### 3.4.2. Time Update

Discretizing the state-space model above, the time update equation in the instant k is

(11) $$\hat{X}(k|k-1)=F(k-1)\hat{X}(k-1|k-1)+G(k-1)W(k-1)$$

where F(k−1) is the discretized transition matrix of the system, G(k−1) is the corresponding system noise matrix, W(k−1) is system noise with a covariance matrix Q(k−1). Let $C_{A}=diag(-\tau_{A}^{-1})$, $C_{G}=diag(-\tau_{G}^{-1})$, and $T_{s}$ be the sampling time, then we get

(12) $$F(k-1)\approx I_{15\times 15}+\left[\begin{array}{ccccc} 0_{3\times 3} & 0_{3\times 3} & 0_{3\times 3} & -C_{b}^{u} & 0_{3\times 3}\\ f^{u}\times & 0_{3\times 3} & 0_{3\times 3} & 0_{3\times 3} & C_{b}^{u}\\ 0_{3\times 3} & I_{3\times 3} & 0_{3\times 3} & 0_{3\times 3} & 0_{3\times 3}\\ 0_{3\times 3} & 0_{3\times 3} & 0_{3\times 3} & C_{G} & 0_{3\times 3}\\ 0_{3\times 3} & 0_{3\times 3} & 0_{3\times 3} & 0_{3\times 3} & C_{A} \end{array}\right]T_{s}$$

(13) $$G(k-1)\approx \left[\begin{array}{cccc} -C_{b}^{u} & 0_{3\times 3} & 0_{3\times 3} & 0_{3\times 3}\\ 0_{3\times 3} & C_{b}^{u} & 0_{3\times 3} & 0_{3\times 3}\\ 0_{3\times 3} & 0_{3\times 3} & 0_{3\times 3} & 0_{3\times 3}\\ 0_{3\times 3} & 0_{3\times 3} & I_{3\times 3} & 0_{3\times 3}\\ 0_{3\times 3} & 0_{3\times 3} & 0_{3\times 3} & I_{3\times 3} \end{array}\right]$$

(14) $$W(k-1)={\left[\begin{array}{cccc} w_{G}^{T} & w_{A}^{T} & w_{rG}^{T} & w_{rA}^{T} \end{array}\right]}^{T}$$

and the covariance matrix propagation equation is

(15) $$P(k|k-1)=F(k-1)P(k-1|k-1)F{\left(k-1\right)}^{T}+G(k-1)Q(k-1)G{\left(k-1\right)}^{T}$$

where P(k|k−1) is the predicted covariance matrix for prior estimation of the state vector. Q(k−1) is the variance-covariance matrix of the state vector.

#### 3.4.3. Measurement Update

The discretized measurement equation of instant k is

(16) $$Z(k)=\left[\begin{array}{c} {\hat{v}}_{imu,k}-{\hat{v}}_{uwb,k}\\ {\hat{P}}_{imu,k}-{\hat{P}}_{uwb,k} \end{array}\right]=H(k)X(k|k)+V(k)$$

where V(k) is the measurement errors vector of the discretized system with a covariance matrix R(k). The matrix H(k) is given by

(17) $$H(k)\approx \left[\begin{array}{ccc} 0_{6\times 3} & I_{6\times 6} & 0_{6\times 6} \end{array}\right]$$

When UWB and IMU data are received, the measurement update is launched. In the measurement update progress, the MCKF updates the posterior estimation by adopting a maximum correntropy (MC) criterion as the optimality criterion. The difference between the MC criterion and minimum mean square error (MMSE) criterion is that higher-order statistical information of the error vector is considered in the MC criterion. One of the cost functions based on the MC criterion is

(18) $$J_{MC}(X(k|k))=\frac{1}{L}\sum_{i=1}^{L}G_{\sigma }(e_{i}(k))=\frac{1}{L}\sum_{i=1}^{L}G_{\sigma }(d_{i}(k)-w_{i}(k)X(k|k))$$

where $G_{\sigma }(\cdot )$ is a shift-invariant Mercer kernel function, one of which is a Gauss kernel function. $e_{i}(k)$ is the difference between $d_{i}(k)$ and $w_{i}(k)X(k|k)$, also the residual error of the extended state-space model of the original system. $d_{i}(k)$ and $w_{i}(k)X(k|k)$ are both variables related to system state vectors and system observation vectors and are defined as [39]. L is the dimension of the extended state-space model. The optimal estimation of state variables can be obtained by optimizing the cost function, that is, solving the equation

(19) $$\frac{\partial J_{MC}(X(k|k))}{\partial X(k|k)}=\sum_{i=1}^{L}\left[G_{\sigma }(e_{i}(k))w_{i}{\left(k\right)}^{T}(d_{i}(k)-w_{i}(k)X(k|k))\right]=0$$

which is equivalent to solving the fixed-point equation (of X(k|k))

(20) $$X(k|k)={\left(\sum_{i=1}^{L}\left[G_{\sigma }(e_{i}(k))w_{i}{\left(k\right)}^{T}w_{i}(k)\right]\right)}^{-1}\left(\sum_{i=1}^{L}\left[G_{\sigma }(e_{i}(k))w_{i}{\left(k\right)}^{T}d_{i}(k)\right]\right)$$

An algorithm of the fixed-point equation is described as follows. First, rewrite the equation as

(21) $$\hat{X}{\left(k|k\right)}_{t+1}=f(\hat{X}{\left(k|k\right)}_{t})$$

where

(22) $$\hat{X}{\left(k|k\right)}_{t+1}=\hat{X}(k|k-1)+\tilde{K}(k)(Z(k)-H(k)\hat{X}(k|k-1))$$

denotes the estimation of the state vector after t iterations, $\tilde{K}(k)$ is the gain matrix after t iterations which is given by

(23) $$\tilde{K}(k)=\tilde{P}(k|k-1)H{\left(k\right)}^{T}{\left(H(k)\tilde{P}(k|k-1)H{\left(k\right)}^{T}+\tilde{R}(k)\right)}^{-1}$$

where $\tilde{P}(k|k-1)$ is the covariance matrix of the state vector correspondingly, $\tilde{R}(k)$ is the covariance matrix of the measurement vector which is obtained based on the MC criterion. When the Equation (23) below does not hold, stop iterating

(24) $$\frac{\left\|\hat{X}{\left(k|k\right)}_{t+1}-\hat{X}{\left(k|k\right)}_{t}\right\|}{\left\|\hat{X}{\left(k|k\right)}_{t}\right\|}\leq \tau$$

and the final posterior estimation of the state vector of instant k is obtained by setting $\hat{X}(k|k)=\hat{X}{\left(k|k\right)}_{t+1}$. τ is a small positive number. Correspondingly, the posterior covariance matrix P(k|k) is given as

(25) $$P(k|k)=(I-\tilde{K}(k)H(k))\tilde{P}(k|k-1){\left(I-\tilde{K}(k)H(k)\right)}^{T}+\tilde{K}(k)\tilde{R}(k)\tilde{K}{\left(k\right)}^{T}$$

#### 3.4.4. Initialization

In order to fuse data from the SINS and UWB system, it is necessary to calculate the initial attitude matrix $C_{b}^{u}(0)$ or the initial Euler angle ϕ(0) of SINS relative to the UWB system. We calculate the initial angle using outputs of the camera, UWB and IMU. Firstly, the IMU data is used to align the *z*-axis of the body frame with the Earth’s gravity vector, then the attitude matrix between the body frame and a certain horizontal frame (h-frame) is obtained, which is $C_{b}^{h}(0)$. Since the UWB frame is also a horizontal frame, only the heading angle may not be zero in the three Euler angles between the h-frame and the UWB frame. Using the displacement vector $r_{visual}$ in the h-frame which is calculated by a visual SLAM system and $r_{uwb}$ in the UWB frame which is calculated by the UWB system, the attitude matrix $C_{h}^{u}(0)$ between the h-frame and the UWB frame can be estimated. Finally, the initial attitude matrix $C_{b}^{u}(0)$ between the body frame and the UWB frame is calculated by

(26) $$C_{b}^{u}(0)=C_{h}^{u}(0)C_{b}^{h}(0)$$

According to (25), the Euler angles between the body frame and the UWB frame can also be calculated.

### 3.5. Extension of the Positioning System

**Figure 4:**
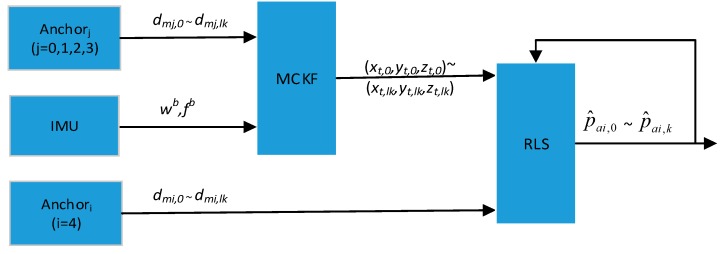
The architecture of the extension system.

When the mobile robot moves to a new environment, it fails to receive the measurement information of anchors which are too far away in the positioning system. It is possible that the mobile robot is unable to get enough positioning information, which leads to positioning failure. Extending the positioning system means adding new anchors to the positioning system. Using the ranging information of the new anchors, the position of the mobile robot can be estimated by the extended system. The architecture of the extension of the positioning system is shown in Figure 4. Firstly, move the mobile robot along a planned trajectory and estimate the positions of the mobile robot. The position $\left(x_{t,lk},y_{t,lk},z_{t,lk}\right)$ of the mobile robot is estimated by an MCKF using the ranging message $d_{mj,lk}$ and IMU measurements $\omega^{b},f^{b}$. The subscript lk denotes the *lk*-th measurement of the variable. Meanwhile, measure the distance $d_{mi,lk}$ of the mobile robot to the anchors that need to add to the positioning system. The subscript mj and mi denote the *j*-th old anchor and *i*-th new anchor. Thirdly, according to Section 3.3, estimate the positions of new anchors using the distances $d_{mi,k}$. The anchors’ positions ${\hat{p}}_{ai,k}$ are optimized by an RLS technic. l measurements are needed to estimate the position of a new anchor at one time, so total lk measurements are needed for k estimations of a new anchor. Finally, add the new anchors to the origin positioning system to extend the system.

To estimate the positions of new anchors, we assume that the positions variables of new anchors obey the Gaussian distribution which can be described as

(27) $$\left\{ \begin{array}{l} {\hat{x}}_{ai}=x_{ai}+\delta x_{ai}\\ {\hat{y}}_{ai}=y_{ai}+\delta y_{ai}\\ {\hat{z}}_{ai}=z_{ai}+\delta z_{ai} \end{array}\right.$$

where $z_{ai}$ is the height of *i*-th new anchor and is known but with an error of $\delta z_{ai}$. $x_{ai}$, $y_{ai}$ and $z_{ai}$ are the true coordination of the new anchor. $\left({\hat{x}}_{ai},{\hat{y}}_{ai},{\hat{z}}_{ai}\right)$ is the estimation of the position of the new anchor. As the position of the new anchor is fixed, the estimation error $\delta x_{ai}$, $\delta y_{ai}$ and $\delta z_{ai}$ are all zero-mean Gaussian white noise sequence. Rewrite the Equation (26) using a simple form as

(28) $${\hat{p}}_{ai}=H_{ai}p_{ai}+\delta p_{ai}$$

where $H_{ai}$ is a matrix with unit diagonal, $\delta p_{ai}$ is the estimation error with a covariance matrix $R_{ai}$. To op optimize the estimation, minimize the cost function below

(29) $$\min\left|\right|\delta p_{ai}\left|\right|=\min\left|\right|{\hat{p}}_{ai}-p_{ai}\left|\right|$$

which can be expanded as

(30) $$\begin{array}{ll} J & ={\left({\tilde{p}}_{ai}-H_{ai}{\hat{p}}_{ai}\right)}^{T}({\tilde{p}}_{ai}-H_{ai}{\hat{p}}_{ai})\\ & ={\tilde{p}}_{ai}^{T}{\tilde{p}}_{ai}-{\hat{p}}_{ai}^{T}H_{ai}^{T}{\tilde{p}}_{ai}-{\tilde{p}}_{ai}^{T}H_{ai}{\hat{p}}_{ai}+{\hat{p}}_{ai}^{T}H_{ai}^{T}H_{ai}{\hat{p}}_{ai} \end{array}$$

where ${\tilde{p}}_{ai}$ is an estimation of the position using l measurements of IMU and UWB respectively. We optimize the estimation though a recursive least squares method. The mentioned recursive least squares method is described as follows

(31) $$K_{ai}(k)=P_{ai}(k-1)H_{ai}^{T}(k){\left(H_{ai}(k)P_{ai}(k-1)H_{ai}^{T}(k)+R_{ai}(k)\right)}^{-1}$$

(32) $${\hat{p}}_{ai}(k)={\hat{p}}_{ai}(k-1)+{\hat{p}}_{ai}K_{ai}(k)({\tilde{p}}_{ai}(k)-H_{ai}(k){\hat{p}}_{ai}(k-1))$$

(33) $$P_{ai}(k)=(I-K_{ai}(k)H_{ai}(k))P_{ai}(k-1){\left(I-K_{ai}(k)H_{ai}(k)\right)}^{T}+K_{ai}(k)R_{ai}(k)K_{ai}^{T}(k)$$

where $P_{ai}(k)$ is the error covariance matrix of ${\hat{p}}_{ai}(k)$, $K_{ai}(k)$ is the gain matrix in instant k, $R_{ai}(k)$ is covariance matrix of measurements which has been described in (27), ${\hat{p}}_{ai}(k)$ is the optimization of the position.

It should be noted that the trajectory of the mobile robot is designed instead of a random trajectory. When the route is circular, the position of anchors can be estimated more quickly and better [45]. Besides, according to [46], the range of movement of the mobile robot needs to be large enough and the distance between the l measurements of the position of the mobile robot used to estimate the position of the new anchor needs to be sufficiently large.

### 3.6. Positioning Using the Extended Positioning System

The extended positioning system contains anchors where the position is not completely determined. It is a very complex problem to locate mobile robots using anchors with uncertain positions. Fortunately, some scholars have studied how to locate tags using anchors with uncertain locations [47]. Referring to the maximum likelihood estimation method, we present a likelihood function with anchors’ positions uncertainty

(34) $$\ln p(d_{mi},{\hat{p}}_{ai,k};p_{t},p_{ai})=-\frac{1}{2}{\left(d_{mi}-f(p_{t},p_{ai})\right)}^{T}C^{-1}(d_{mi}-f(p_{t},p_{ai}))+\sum_{i=1}^{N}\left(\frac{{\left(\left\|{\hat{p}}_{ai,k,i}-p_{ai,i}\right\|\right)}^{2}}{2\sigma_{\alpha }^{2}}\right)$$

where C is a covariance matrix of vector $\left[f(p_{t},p_{a0}),f(p_{t},p_{a1}),\cdots ,f(p_{t},p_{ai})\right]$, $f(p_{t},p_{ai})$ is a ranging which considers ranging errors between the tag’s position $p_{t}$ and the *i*-th anchor’s position $p_{ai}$, $d_{mi}$ is a vector of ranging of UWB system, $p_{ai,i}$ is the mean of anchor’ positions ${\hat{p}}_{ai,k,i}$ which obey the Gaussian distribution, $\sigma_{\alpha }^{2}$ is the variance correspondingly.

Since we have enough measurement of the position of the anchors, the final estimation of the anchor’s position is sufficiently accurate. Therefore, we consider all anchors’ locations in the extended positioning system to be known exactly. This simplifies the positioning of the system. Another reason is that in our system, the output of the IMU on the mobile robot can also be used to assist in estimating the position of the mobile robot. It can be seen from the experiment that the positioning result of the mobile robot using this method is acceptable. Based on the above considerations, we use the method presented in Section 3.4 to estimate the position of the mobile robot after extending the positioning system.

To mimic the positioning of robots in unfamiliar environments, we assume that some anchors are not available when the robot moves to an unfamiliar environment. In the extended positioning system, the newly added anchors can be detected by the mobile robot. However, some older anchors, or anchors that are farther away from the mobile robot, may not be detected by the mobile robot. If some anchors are lost, the system uses the last few anchors which can be found by the tag. If all of the anchors are lost, the system will be replaced by SINS (failure mode). However, we assume that at least four anchors can be found by the tag to provide adequate ranging information. Therefore, the extended positioning system is able to provide enough ranging information.

## 4. Experiment and Discussion

**Figure 5:**
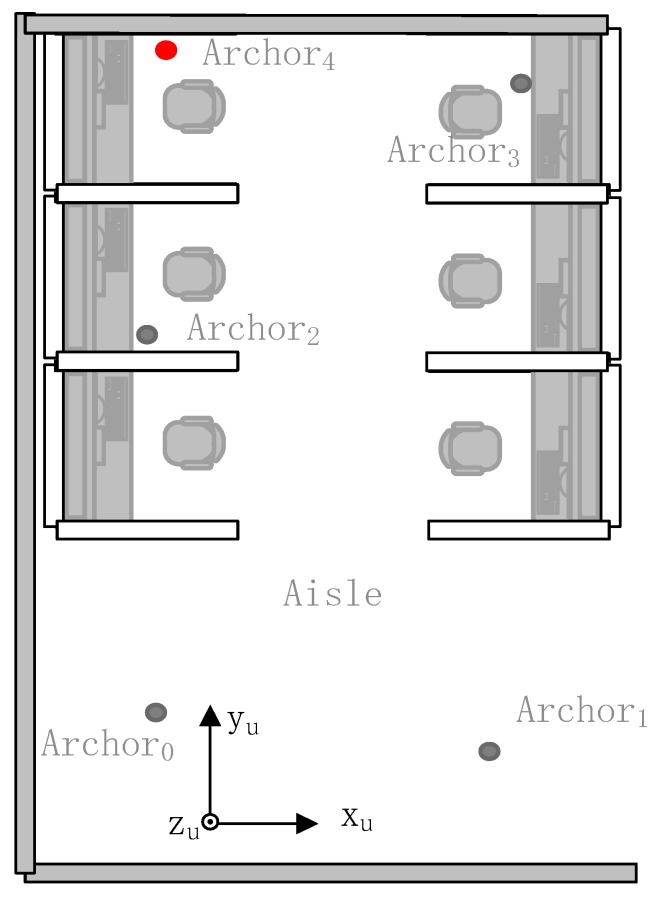
Experimental site.

The plan of the experimental site and the location of the anchors are shown in Figure 5. In this paper, the experimental site is a school laboratory (a $3.5\times 9(m^{2})$ laboratory) which includes all kinds of obstacles. In the experiment, four anchors ${\mathrm{Anchor}}_{j}(j=0,1,2,3)$ which are black anchors in the figure are known and are parts of the original positioning system. The positions of these anchors are considered to be accurately calibrated by a laser rangefinder. One anchor ${\mathrm{Anchor}}_{i}(i=4)$ which is the red anchor in the figure is the new anchor that is used to extend the positioning system. The position of the red anchor is unknown. The coordinate shown in Figure 5 is the UWB frame, and it is introduced in Section 3. The coordinates of the anchors are shown in Table 1. We measure the positions of anchors by using a laser rangefinder. The measurement parameters of the laser rangefinder and the UWB transceivers are given in Table 2. The ranging error of UWB transceivers which is shown in Table 2 is estimated under a favorable condition which is called light-of-sight (LOS) condition. When there are many obstacles in the environment of the positioning which is the exact situation in our experiment, the ranging error changes.

We use the result of a SLAM positioning system as a reference path of our mobile robot. The SLAM positioning system based on key-frames uses a stereo camera which is introduced before to locate the mobile robot. The stereo camera which is shown in Figure 1 integrates a low-cost IMU. To evaluate the performance of our approach, we compare the estimated path with the reference path.

The whole experiment includes four parts. In the first part, we use the positioning system to locate the mobile robot using four anchors of which the positions are known. Then, in the second part, we estimate the position of a new anchor of which the position is considered unknown. In the third part, an extended positioning system which includes the original system is used to locate the mobile robot. Then, in the last part, we consider that one anchor of the positioning system is lost to simulate the situation where the mobile robot moves into an unfamiliar environment.

### 4.1. Positioning for the Mobile Robot

**Figure 6:**
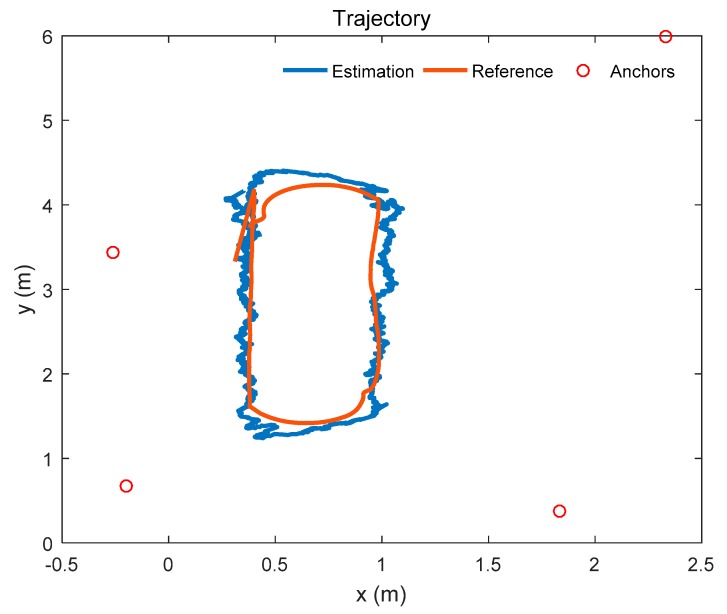
Trajectory of the mobile robot.

The trajectory of the mobile robot is shown in Figure 6. The mobile robot moves along the o-shaped trajectory. The red trajectory is the reference path, and the blue trajectory is the estimated path which is given by the positioning system. In this experiment, only 4 anchors are used in the system. The anchors are shown in Figure 6, and are represented by the red circles in the figure.

**Figure 7:**
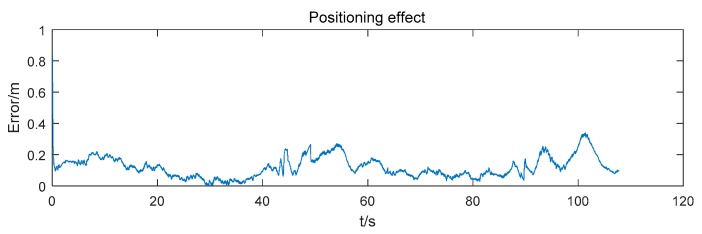
Positioning error using 4 anchors.

The positioning error of the mobile robot is shown in Figure 7. At first, the positioning error decreases rapidly. Then, as time goes on, the positioning error convergences gradually. The root mean square error (RMSE) of the positioning result is 0.137 m.

### 4.2. Adding a New Anchor

**Figure 8:**
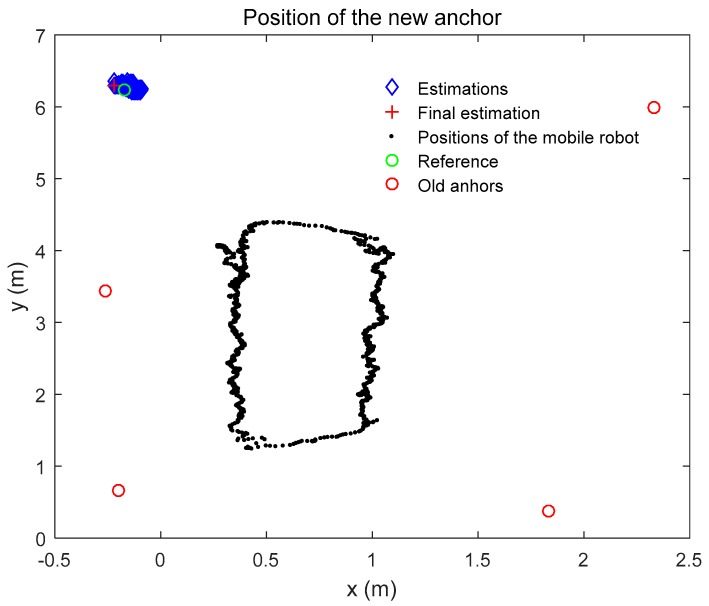
Positioning of the new anchor.

While positioning the mobile robot, a new anchor is added to the system to extend the system itself. By using the ranging between the mobile robot and the new anchor, the position of the new anchor is estimated by the RLS approach. The positions of the mobile robot are estimated before. The ranging distances are measured while the mobile robot moves along the trajectory. The position estimation of the new anchor is shown in Figure 8.

**Figure 9:**
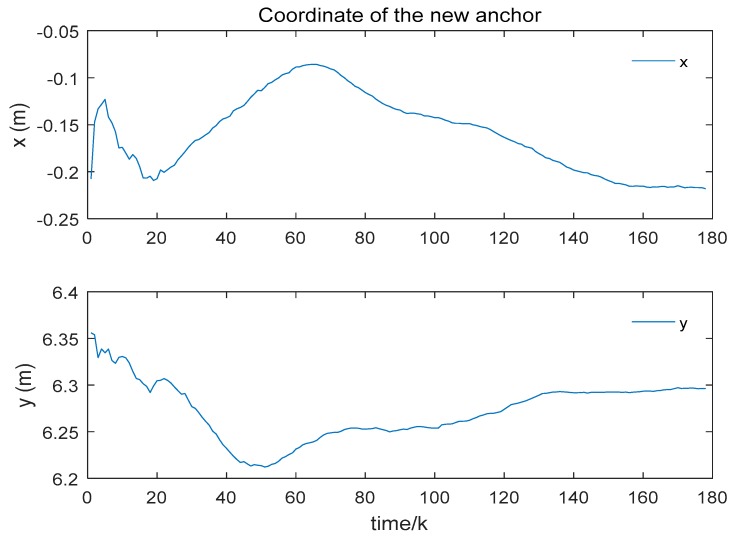
Coordinate estimation of the new anchor.

We give the coordinate estimations of the new anchor in Figure 9. As shown in the figure, the coordinate of the new anchor is close to the reference coordinate which is measured by the laser rangefinder. The reference coordinate (*x*, *y*) of the new anchor is given in Table 3. The height (*z*) of the new anchor is known as a true value. The final position estimation of the new anchor is shown in Table 3 as well. Note that the errors presented are different in two axes. As the trajectory of the mobile robot is not a circle or the coordinate difference of the old anchors in *x*-axis is different from that in *y*-axis. As shown in Table 3, the RMSE of Euler distance error is 0.061 m. In addition, we calculate the coordinate of the new anchor with four methods. The results of the calculation are also shown in Table 3. The label “MCKF-RLS” represents that we estimate the positions of the mobile robot using the MCKF and then estimate the position of the new anchor with the RLS. In Table 3, It indicates that all of the methods are able to locate the position of the new anchor using the proposed scheme which suggests the reasonability of our scheme. Besides, it shows that the RMSE of Euler distance using MCKF-RLS is better than the other methods while the other types of results are comparable with the other approaches. This illustrates the effectiveness of our strategy.

### 4.3. Positioning Using the Extended Positioning System

**Figure 10:**
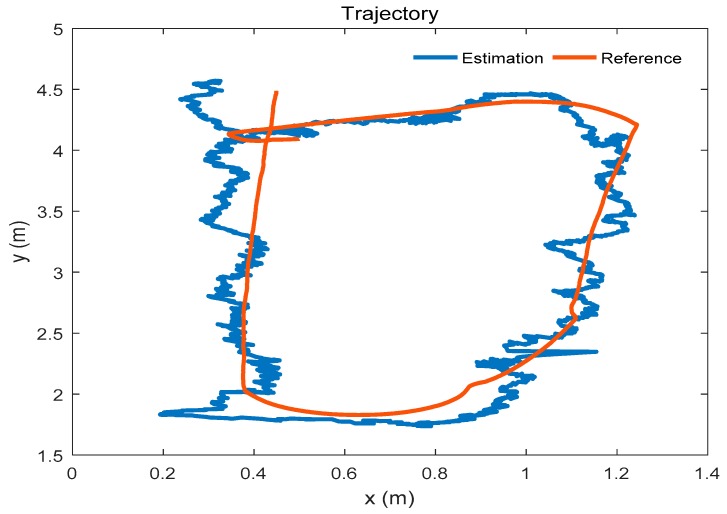
Positioning using the extended positioning system.

**Figure 11:**
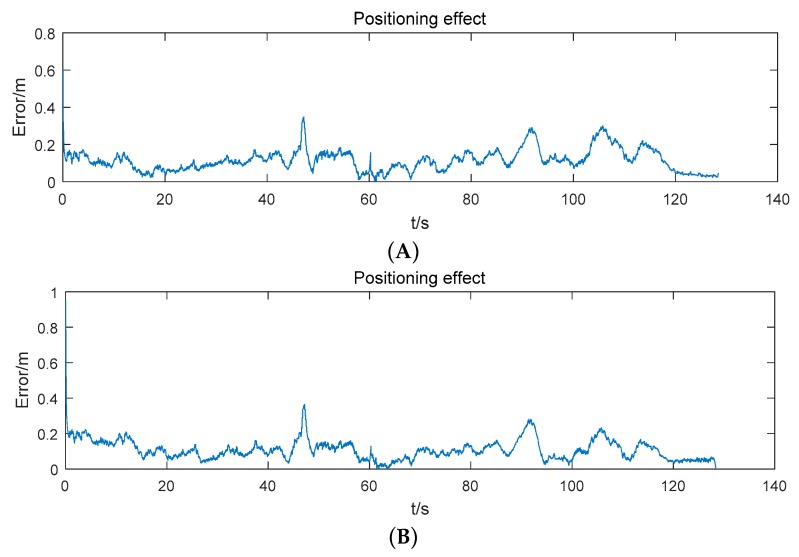
Positioning effect of the positioning system. (**A**) Using the estimation position of the new anchor. (**B**) Using the reference position of the new anchor.

After extending the system by adding the new anchor, we use the extended positioning system to locate the mobile robot. The mobile robot moves in the school laboratory. The trajectory which is estimated by the extended positioning system is shown in Figure 10. The positioning error of the mobile robot is given in Table 4 and Figure 11. As a comparison, we use a reference positioning system which includes all anchors to locate the mobile robot. The positions of the anchors in the reference positioning system are measured by the laser rangefinder. The positioning error of the mobile robot and the difference of the RMSE given by the two systems is shown in Table 4 and Figure 11 as well.

As shown in Table 4 and Figure 11, the positioning effect of the extended positioning system is close to that given by the reference system which uses all reference positions of all anchors. It means that the estimation of the extended system is credible and it is reliable to replace the reference system using the extended positioning system.

### 4.4. Positioning in an Unfamiliar Environment

**Figure 12:**
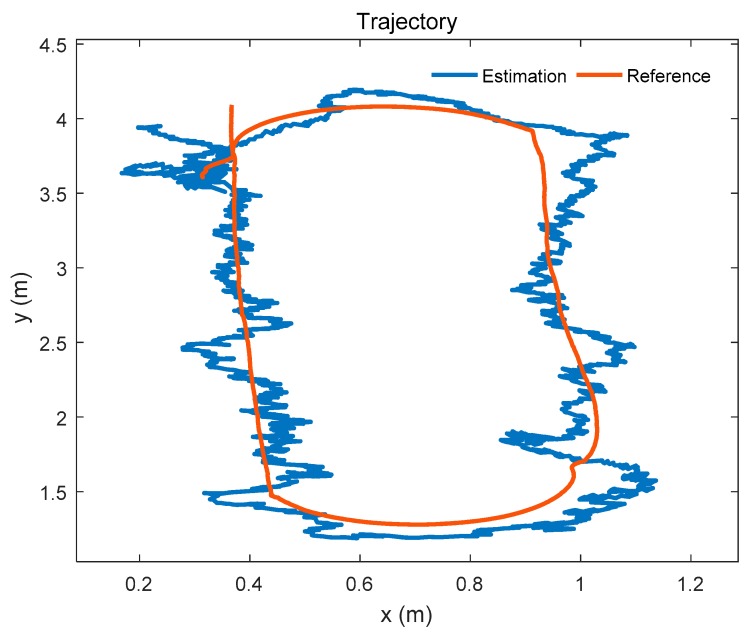
Positioning in an unfamiliar environment.

To simulate the unfamiliar environment where the UWB anchor is lost, we consider that the Anchor_0_ loses contact with the mobile robot. In this case, the number of available anchors of the extended positioning system is four. And the positions of remaining anchors are considered as known. A trajectory of the mobile robot is shown in Figure 12.

**Figure 13:**
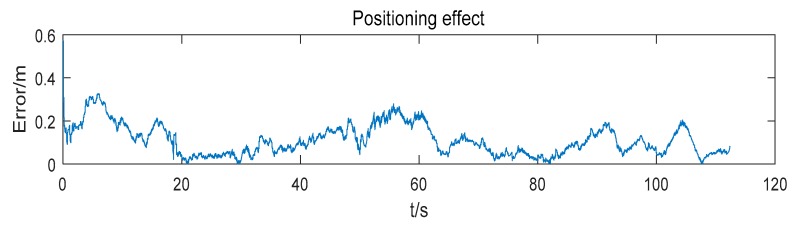
Positioning effect in an unfamiliar environment.

The positioning error of the mobile robot is shown in Figure 13. The RMSE of the position estimation is 0.131 m. The positioning effect of the mobile robot using the extended positioning system is close to Figure 7 which uses the reference system with four anchors (the difference of the RMSE between two systems is 0.006 m). Although the positioning effect is a little worse than the reference system and the extended system which includes all anchors (the difference between two results is 0.008 m), it is acceptable.

In order to show the performance of the extended system clearly, we compare the results obtained by three different methods. The results of the three methods are given in Table 5. It shows that the RMSE given by the extended system using four anchors (the extended system used in an unfamiliar environment, ES-UE) is close to that given by the original system, and the performance of the extended system using all anchors is close to that of the reference system. Thus, the extended system is able to replace the original system or the reference system. In addition, the RMSE given by the MCKF fusion is smaller than the other methods in most of the environments. It suggests the effectiveness of the MCKF fusion and our scheme. Besides, the results of the mean and standard deviation (Std) which is based on the absolute value of the errors are given in Table 5.

## 5. Conclusions

The paper presents an extensible positioning system to locate the mobile robot with a radius of 25 cm and a height of 25 cm which is equipped with an IMU, a UWB tag, a Stereo camera module and a WiFi router. The positioning system estimates the positions of the mobile robot by using the ranging of UWB transceivers and the angular velocity and linear velocity measured by the IMU. The system fuses data by an MCKF approach which is based on an MC criterion. By locating the mobile robot using the original system, and then estimating the position of a new anchor using the ranging between the mobile robot and the new anchor, the positioning system is extended after adding the new anchor to the original system. In this scheme, the positioning system is without the requirement of communication between anchors and the requirement clock synchronization of anchors. It estimates the initial attitude of the mobile robot relative to the navigation frame by comparing estimations of the same displacement vectors given by the UWB system and a SLAM system, respectively. In an unfamiliar environment of the mobile robot, the extended positioning system is able to estimate the position of the mobile robot by adding new anchors. The RMSE of the positioning result is 0.131 m (the experiment site is a $3.5\times 9(m^{2})$ laboratory) while using the extended positioning system (unfamiliar environment) which is closed to the result given by the original system. On the other hand, the results given by the extended system are close to that of the reference system (all anchors). Besides, the RMSE of the new anchor presented in Euler distance is 0.061 m. However, some other experiments may be needed to make sure that the system is fit for a larger environment.

An improvement of the system may be found by using an MDS approach to reduce the position uncertainty of anchors and to consider the transmission error nature of the UWB transceiver signal by using Equation (34). Besides, the future work of the paper is to integrate the visual SLAM system into the whole system to improve the robustness and performance of the system.

## Figures and Tables

**Table 1:** Coordinates of anchors.

Anchors	Coordinates (*x*, *y*, *z*) (m)
Anchors_0_	(−0.197, 0.671, 1.8)
Anchors_1_	(1.836, 0.375, 2.0)
Anchors_2_	(−0.262, 3.440, 1.6)
Anchors_3_	(2.330, 5.995, 2.0)
Anchors_4_	(−0.174, 6.242, 2.3)

**Table 2:** Measurement parameters of modules.

Module	Measurement Error (m)
Laser rangefinder.	<0.005
UWB transceivers	<0.15

**Table 3:** Coordinate of the new anchor.

Type	Value of (*x*, *y*) (m)			
	MCKF-RLS	KF-RLS	UWB-RLS	UWB-LS
Reference	(−0.197, 6.242)	(−0.197, 6.242)	(−0.197, 6.242)	(−0.197, 6.242)
Final estimation	(−0.218, 6.296)	(−0.225, 6.304)	(−0.181, 6.245)	(−0.399, 6.166)
RMSE	(0.043, 0.043)	(0.041, 0.053)	(0.064, 0.024)	(0.131, 0.101)
RMSE (distance)	0.061	0.067	0.068	0.166
Mean	(−0.160, 6.272)	(−0.167, 6.283)	(−0.125, 6.232)	(−0.180, 6.246)
Standard deviation	(0.041, 0.031)	(0.041, 0.033)	(0.039, 0.022)	(0.132, 0.101)

**Table 4:** Positioning error using the extended system.

System	RMSE (m)	Mean (m)	Standard Deviation (Std) (m)
Original positioning system (OPS)	0.137	0.119	0.067
Reference positioning system (RPS)	0.122	0.116	0.058
Extended positioning system (EPS)	0.130	0.108	0.057
Difference between RPS and EPS	0.008	0.008	0.001

**Table 5:** Positioning error using difference methods.

System	Positioning Performance (m)								
	MCKF Fusion			KF Fusion			UWB Only		
	RMSE	Mean	Std	RMSE	Mean	Std	RMSE	Mean	Std
Original system	0.137	0.119	0.067	0.144	0.121	0.078	0.160	0.149	0.058
Extended system	0.130	0.116	0.058	0.136	0.122	0.061	0.154	0.141	0.061
Reference system	0.122	0.108	0.057	0.126	0.113	0.055	0.142	0.130	0.057
ES-UE	0.131	0.111	0.069	0.140	0.121	0.072	0.171	0.155	0.072
